# “I would rather be told than not know” - A qualitative study exploring parental views on identifying the future risk of childhood overweight and obesity during infancy

**DOI:** 10.1186/s12889-017-4684-y

**Published:** 2017-08-29

**Authors:** Faye Bentley, Judy Anne Swift, Rachel Cook, Sarah A Redsell

**Affiliations:** 1 Anglia Ruskin University, Cambridge, United Kingdom; 2 Behavioural Nutrition, University of Nottingham, Nottingham, United Kingdom; 3 Department of Psychology, Anglia Ruskin University, Cambridge, United Kingdom; 4 Faculty of Health, Social Care and Education, Anglia Ruskin University, Cambridge, United Kingdom

**Keywords:** Infant, Childhood, Overweight, Obesity, Parents, Feeding, Obesity risk, Risk communication

## Abstract

**Background:**

Risk assessment tools provide an opportunity to prevent childhood overweight and obesity through early identification and intervention to influence infant feeding practices. Engaging parents of infants is paramount for success however; the literature suggests there is uncertainty surrounding the use of such tools with concerns about stigmatisation, labelling and expressions of parental guilt. This study explores parents’ views on identifying future risk of childhood overweight and obesity during infancy and communicating risk to parents.

**Methods:**

Semi-structured qualitative interviews were conducted with 23 parents and inductive, interpretive and thematic analysis performed.

**Results:**

Three main themes emerged from the data: 1) Identification of infant overweight and obesity risk. Parents were hesitant about health professionals identifying infant overweight as believed they would recognise this for themselves, in addition parents feared judgement from health professionals. Identification of future obesity risk during infancy was viewed positively however the use of a non-judgemental communication style was viewed as imperative. 2) Consequences of infant overweight. Parents expressed immediate anxieties about the impact of excess weight on infant ability to start walking. Parents were aware of the progressive nature of childhood obesity however, did not view overweight as a significant problem until the infant could walk as viewed this as a point when any excess weight would be lost due to increased energy expenditure. 3) Parental attributions of causality, responsibility, and control. Parents articulated a high level of personal responsibility for preventing and controlling overweight during infancy, which translated into self-blame. Parents attributed infant overweight to overfeeding however articulated a reluctance to modify infant feeding practices prior to weaning.

**Conclusion:**

This is the first study to explore the use of obesity risk tools in clinical practice, the findings suggest that identification, and communication of future overweight and obesity risk is acceptable to parents of infants. Despite this positive response, findings suggest that parents’ acceptance to identification of risk and implementation of behaviour change is time specific. The apparent level of parental responsibility, fear of judgement and self-blame also highlights the importance of health professionals approach to personalised risk communication so feelings of self-blame are negated and stigmatisation avoided.

## Background

Worldwide, 42 million infants and young children are overweight or obese [1]. The escalating prevalence of obesity in pre-school children is a growing global public health concern and early intervention is urgently required to reverse anticipated trends. Systematic reviews have identified a number of factors in pregnancy and early infancy that are associated with an increased risk of overweight in childhood [2, 3]. The identification of these factors and the development of childhood overweight and obesity risk prediction tools [4–8] provide a tangible opportunity for early intervention.

Effective communication of risk is an essential step in engaging parents in childhood obesity prevention. However, there are practical and ethical issues associated with the use of risk prediction tools in clinical practice [9, 10]. Ethical concerns include the possible unintended psychosocial consequences of intervention upon parents and children, such as stigmatisation, blame and parental guilt [11]. Existing research in this area suggests there is parental uncertainty about the identification of future risk of overweight during infancy, as well as specific anxieties about stigma and labelling of young children [4, 12]. Furthermore, recent research indicates that the provision of obesity risk information may cause parental guilt [13].

The failure of parents to recognise an unhealthy weight status within their own child has been suggested as an explanation for poor parental engagement in interventions to prevent and manage childhood obesity [14–16]. Evidence also suggests that parents lack awareness of the impact of childhood overweight upon health [17] and that a higher value is given to on other attributes such as childhood happiness [18].

An improved understanding of parental views about overweight and obesity during infancy is crucial to unravelling the motivations and barriers to engaging parents in early obesity prevention efforts and influencing behaviour change. The purpose of this this is to understand the views of parents of infants on assessing and communicating the future risk of childhood overweight and obesity. It explores parental perceptions of the factors associated with the development of excess weight during infancy, beliefs surrounding the prevention and management of infant overweight and views on a hypothetical intervention in which the future obesity risk is predicted using a risk prediction tool and communicated to parents.

## Methods

### Population

The sample was drawn from families living in Cambridgeshire, with infants aged 12 months or younger who were attending existing mother and baby groups or weighing clinics at Sure Start Children’s Centres [19]. The three Children’s Centres from which parents were recruited were selected as sites due to their geographical location within the local authority wards of high deprivation identified by Index of Multiple Deprivation [20]. Obesity prevalence rates amongst children in reception year children (age 4-5 years) compared to Cambridgeshire as a county were also used to inform selection of study sites using data from the National Child Measurement Programme (NCMP) [21].

### Sample

The study population were parents and guardians of infants 12 months of age or younger. Parents were excluded if they were unable to understand spoken English, as funding was not available for interpreting and translation services.

### Sample Size

An adequate sample size was determined as the point at which saturation was reached and that to continue interviewing would have no further benefit to the depth and diversity of the data collection [22].

### Recruitment

Three of the four Children’s Centres approached agreed to participate. The lead author attended four existing groups for parents of children aged 12 months or less to recruit parents. Three of these groups were mother and baby groups and the fourth was a drop-in weighing clinic. Parents were provided with a verbal explanation of the study, supported by a written information sheet, and an opportunity to ask questions. Parents wishing to participate provided their contact details and written consent prior to leaving the children’s centre and then a mutually convenient time and place (either at their home or at the Children’s Centre) for the interview was arranged.

### Data collection

Face-to-face interviews took place in summer 2014. These were conducted using a semi-structured interview guide (Table 1) informed by existing research [12]. After the interview, participants were invited to complete a structured questionnaire collecting self-reported data on parental (age, gender, ethnicity, employment status, level of education, smoking status, and weight and height) and infant (age, gender, birth weight, and breastfeeding status) characteristics.

**Table 1:** Topic Guide

To investigate parental perceptions on the factors associated with the development of excess weight in infancy.
Please tell me about your baby’s growth since birth, have you ever had any concerns or worries about their growth?
Do you think you would be concerned if you thought your baby was gaining weight too quickly?
What do you think could be some of the reasons why a baby might gain excess weight? [Expand by asking why these factors, how important factors are in relation to each other]
Discussion and opinion about other reasons
How much control do you think a parent has over their baby’s weight?
Are you aware of any other factors that you think may be important and related to a child becoming overweight?
To explore parents’ perceptions on the solutions to, and prevention of overweight in infancy.
What do you think could be done to help an infant from gaining weight too quickly?
Who needs to be involved in helping to prevent children becoming overweight/to help children remain a healthy weight? Whose responsibility do you think it is to intervene?
How much control do you think parents have in preventing children from becoming too large/big, when they are a baby? Have you ever felt or have known other parents that have felt blamed when their babies/children become overweight? Where does the blame come from?
To explore parents’ beliefs on the benefits, harms of and emotional response to the idea of an intervention in which the future risk of their infant developing childhood obesity is identified and communicated.
If a Dr./Health Visitor was able to assess a babies future risk of becoming an overweight child do you think they should do this?
Who should they tell/why? What should they say? How would it be best explained
As a parent, what might be the benefits of knowing this information?
What concerns do you think you might have as a parent?
What support do you think you would like or need having been told about this?

### Data analysis

Interviews were audio recorded, transcribed verbatim, and entered into QSR NVIVO Version 10 (Sage Publications Software). The lead author then employed an inductive, interpretative thematic analysis [23]. All members of the research team were involved in developing themes by discussing convergence, divergence, and context. Themes are presented with verbatim quotes in italics.

## Results

### Participant information

The lead author attended the Children’s centre groups on seven occasions to recruit parents. Forty four parents were approached, of these, 36 consented and 20 went on to be interviewed (3 interviews involved both parents) (Table 2). Reasons for non-response following initial interest included, no answer to calls/no response to voice mail messages or wrong number provided by participants (*n* = 7), withdrawal due to family bereavement (*n* = 1), lack of time (*n* = 1), feeling uncomfortable about the subject matter (*n* = 1), or no reason stated (*n* = 3), and the baby was >12 months (*n* = 1). One interview was excluded due to an incomplete audio-recording. Twelve interviews took place at a Children’s Centre and eight in the participants’ homes.

**Table 2:** Parent and infant characteristics

Characteristics	n (20)
Age of parent (years)	
20–29	11
30–39	9
Gender	
Female	19
Male	1
Ethnicity	
White British or Irish	18
European	2
Employment status	
Unemployed	5
Employed	14
Self-employed	1
Smoking status	
Regular smoker	3
Non smoker	13
Occasional smoker	4
Parent Body Mass Index (BMI)/weight status	
< 18.5 (Underweight)	1
18.5–24.9 (Healthy Weight)	7
25–29.9 (Overweight)	7
30–39.9 (Obese)	3
40 > (Morbidly Obese)	1
Missing Data	1
Level of education	
GCSEs	3
NVQs	2
A Level	3
Diploma	7
Degree	3
Higher Degree	2
Age of infant	
Newborn – 12 weeks	1
3–6 months	13
7–9 months	3
10–12 months	3
Infant gender	
Male	10
Female	10
Infant Birth Weight	
< 2.93 kg	4
2.93 to <3.24 kg	5
3.24 to <3.49 kg	2
3.49 kg to <3.81 kg	5
> 3.81 kg	4
Infant ever breastfed	
Yes	18
No	2
Geographical Area of Cambridgeshire	
Wisbech	16
Littleport	3
Ely	1

### Interviews

Three main themes and associated subthemes emerged from the interview data (Table 3).

**Table 3:** Themes and sub-themes from the data analysis

Main themes	1) Identification of infant overweight and future risk	2) Consequences of infant overweight status	3) Parental attributions of causality, responsibility and control
Sub-themes	1a) Overweight as a concept that can be applied to infants	2a) The relative impact of overweight versus underweight	3a) High parental responsibility for overfeeding
	1b) Trust and mistrust of professional growth assessment	2b) The progressive nature of consequences associated with overweight	3b) Low parental self-efficacy for modifying infant feeding
	1c) Receptiveness to risk communication and modification of lifestyle behaviours	2c) The importance of infant contentment	
	1d) Perceived benefits and harms of risk communication	2d) Good parenting and the fear of negative judgement	

### Theme 1: Identification of infant overweight and future risk

#### Subtheme 1a: Overweight as a concept that can be applied to infants

Participants readily accepted childhood obesity as a prevalent and significant issue within society, and did not challenge the idea that a conversation around infant overweight was appropriate.

“… the future is looking fat, isn’t it?” (P10).

Parents replied with little hesitation that they would be able to recognise overweight or rapid growth in their baby, justifying this as a matter of common sense and utilising norm-referencing and comparison to peers.

Well I think it is just a matter of common sense if they look really fat (P7).

“When I go to visit my friends and I saw two or three week old babies and they were smaller than mine (when he) was born just now it was oh he’s a big boy. And now he is big too because now he’s seven and half kilo around something, so it’s really big because he is just three and a half months, like my friend have a girl and she is eight months and just eight kilos” (P20).

Participants generally used socially constructed comparisons, to emphasise shape and form as descriptors by making visual comparisons to siblings and peers, or being able to fit into clothing with an appropriate age-label.

“He’s wearing one- to two-year-old clothes and he is not even one yet” (P16).

Although not explicitly asked to (as per the interview schedule), some parents went on to describe their own infant as overweight using terms ranging from overweight and obese, to big, massive, and chubby, and also chunky monkey.

#### Subtheme 1b: Trust and mistrust of professional growth assessment

Despite the positive attitude to self-identification, parents demonstrated hesitation about the role of health professionals in identifying infants as overweight.

“The first responsibility it’s with the parent but there should be some professional help …{to}…help parents recognise that the baby is becoming overweight” (P18).

Partly this was because it was felt to be unnecessary, since they as parents could be relied upon to identify it, but also due to experience of, or concerns about, being judged.

“I don’t think parents should need to be intervened because they should be able to see their child is obese…” (P14).

“..You wouldn’t be happy because you would feel like he was sectioned out from all the other babies … people would be saying oh that baby is fat, you wouldn’t be very happy.” (P15).

Parents were very aware of the growth charts and centiles used by health professionals, and referred to them as charts, the red book, or the line. However, accounts suggest some uncertainty about what the centile lines represent as well as widespread scepticism around the growth standards.

“In our baby’s health book um and the lines go from 0.4% all the way through to, well I think it’s through to 100% it might even be more than that and I’m guessing that your aiming for the 50^th^ percentile on that um but nobody has ever really said anything about it…” (P1).

“But I think the line is pretty stupid anyway if I’m honest, because each child is individual and they will grow as they want” (P14).

Notably, some participants described circumstances in which the growth standards did not apply to them.

“Because she was born so heavy she didn’t seem to work so well with the growth charts in the little red book” (P7).

Despite lack of enthusiasm for growth standards, parents frequently attended baby-weighing clinics – a process described as checking – and were reassured when their baby was on the line or following the line.

“I mean I do look at him, everyone looks at him and says god he’s massive, he’s only six months and I say when you weigh him and put him on the chart he’s not, he’s bang on the middle…” (P12).

#### Subtheme 1c: Receptiveness to risk communication and modification of lifestyle behaviours

Notably, the possibility of predicting future obesity risk was treated with pleasant surprise and elicited very little questioning from parents. There was an overwhelmingly positive desire to receive information about both their infants’ current weight status, if it was a problem, and the future risk of their infant becoming overweight.

“…I would be happy for her to say it outright if that was a chance then so if she was to say that would be fine” (P13).

“Yeah definitely, definitely if there was something that could be done, or that I was doing wrong that I could do differently then, yeah I would want to know” (P8).

Provision of information about overweight risk was viewed as more acceptable or ‘believable’ when provided once an infant is walking.

“Well you have your two year check, so a two year check would probably be a good one because they are definitely walking then” (P12).

Walking was associated with an increase in energy expenditure and viewed as an important point at which those children with puppy fat (which can be naturally grown out of), are separated from those that will have persistent overweight tracking through life. The degree of parental concern about infant overweight status was linked to an infant’s stage of development. Participants repeatedly suggested that the point at which they would become concerned was when their infant remained overweight once walking.

“…my opinion is that when he starts walking about he’ll burn it off and I’m quite an active person, I walk pretty much everywhere unless isn’t in walking distance and so he will, it will come off him and he will be fine.” (P4).

#### Subtheme 1d: Perceived benefits and harms of risk communication

The primary benefit parents ascribed to knowing about their infant’s future risk was because it acted as a cue to action, with some parents specifying possible behavioural strategies.

“Well at least then you could sort things out a lot quicker…” (P2).

“I suppose if she started to become fat I’d probably think about the amount of calories we are giving her and giving her more vegetables and things that will give her nutrients…” (P7).

“..If I was told he was overweight I would probably be feed him fruit and making him go healthy …” (P14).

However, the possibility of negative behavioural change was raised.

“It would worry me because some mums might panic that their child is going to become overweight as a baby and not feed them as much” (P17).

When questioned about how they might feel after receiving risk information, parents focused on negative feelings; bad parent, ashamed, upset, worried, annoyed, offended, and shocked. Interestingly, participants were not only concerned about feeling guilty for serving their child’s best interests, but also by how this would be perceived (and judged negatively) by others. However, for parents in this study, the potential negative emotions although recognised did not supersede the desire for knowledge.

“… I said I’d feel ashamed, like you know, but I would rather be told I would rather know than not know” (P5).

Finally, the importance of a non-judgemental communication style to reduce negative emotions was identified.

“… not being so abrupt and like out right blaming the parents I think it needs to be done you know in a sensitive way because some parents will, aren’t going to be happy with the fact that you know…” (P17).

### Theme 2 – The consequences of infant overweight status

#### Subtheme 2a: The relative impact of overweight vs. underweight

Concerns about underweight or poor growth were evident and often regarded as more worrying than overweight. High levels of anxiety were articulated about poor infant growth or falling short of the line (see subtheme 1b), which were felt to be caused by the response of alarmist healthcare professionals.

“… they were like we have to get the weight on we have to get the weight on so I found it traumatic for at least the first two weeks” (P6).

#### Subtheme 2b: The progressive nature of consequences associated with overweight

Overwhelmingly parents problematised infant overweight in terms of the likely continuation of excessive weight from infancy into both childhood- and adulthood.

“…I think it’s a real concern, I mean you do not want an overweight child and the concern, I suppose the concern is that if you have an overweight baby they become an overweight child” (P1).

The immediate concerns relating to overweight were in relation to initiation of walking and other developmental milestones.

“…..Just that she’s going to get really obese and by the time she’s a year old not be able to walk because she’s going to be too fat to lift her own weight..” (P19).

Intermediate concerns related to bullying at school, and it was only in relation to adult overweight that health consequences, most notably diabetes and heart attacks, were referenced.

“…I am not fussed at the moment, but when he starts going to nursery or starts going to school then I wouldn’t want him to be overweight at that age.” (P2).

“I’ll try to prevent illness, because I know that being overweight you’ve got a risk of so many like, so many things, arteries, heart attacks in the future and so I know the side effects of being overweight” (P4).

#### Subtheme 2c: The importance of infant contentment

For some the goal of infant contentment over-rode concerns about overfeeding and overweight. Interestingly, participants most frequently discussed a baby’s contentment in the context of regular, ample feeding, and maternal happiness was centred on infant happiness.

“Obviously I think he is pretty chubby, but I don’t see a problem with it because he is happy so” (P15).

“I don’t know, because I don’t know I suppose like if a baby is hungry then a baby is hungry and to sort of reduce their milk if they are still hungry that seems really cruel” (P13).

#### Subtheme 2d: Good parenting and fear of negative judgement

Participants repeatedly voiced concerns about being judged as a bad parent by others for having an overweight child, and some reported experiences of this. When asked about from whom they feared judgement, other parents and people in the street were typical responses.

“As she gets older, and obviously whatever people are going to think about me as a parent for letting her get like it, if she’s like ridiculously overweight or anything like that” (P11).

“I don’t know really just like random people, if you’re walking down the road or something you don’t want someone to shout out, oi your baby’s fat” (P15).

Societal stigma surrounding adult obesity was also apparent in parent narratives, particularly amongst participants who were themselves overweight.

“Yeah they think a fat girl with a fat baby” (P2).

### Theme 3 – Parental attributions of causality, responsibility, and control.

#### Subtheme 3a: High parental responsibility for overfeeding

When questioned about preventing the onset of excess weight gain during infancy all participants stated - without hesitation - that it was their responsibility as parents, and cited overfeeding as the main contributing factor.

“I have all of it, I have all the control at the end of the day I am the one, she is not feeding herself at the moment…” (P17).

“I have not really thought about weight I think it is, the only thing I think it really could be at the moment is overfeeding, feeding when it’s not necessary” (P17).

Overfeeding was discussed primarily in relation to formula milk and solid foods as they described how breastfed babies could not be overweight or, if they were, that this was not unhealthy and would naturally resolve itself.

“If you’re breastfed you can’t, you can’t over eat so if they are gaining weight that is their natural …” (P6).

The sense of responsibility for overfeeding translated into self-blame for parents describing their babies as overweight, which contrasts with the general ease of self-identification (subtheme 1a).

“...I do feel the blame is solely on yourselves because you’re looking after um, you’re the one that’s giving them food and letting them eat it” (P5).

#### Subtheme 3b: Low parental self-efficacy for modifying infant feeding

Contrasting with the sense of parental responsibility, participants voiced a limited sense of control regarding weight-related behaviours. When (voluntarily) disclosing that their baby was overweight, participants went on to comment upon their child’s eating behaviour and openly acknowledged their child had a big appetite or was a hungry baby.

“…so she was just, ate more and more and more, the more you offer the more she will have” (P19).

Although parents did not use terminology such as responsive feeding, they distinguished feeding cues and described using strategies such distraction, the use of a dummy, and water to reduce the frequency of feeds. However, there was also a deep reluctance to implement any changes to their child’s milk diet beyond what they perceived their infant needed, regardless of any effect on weight.

“…I think if she is hungry then she is hungry … they obviously say that they need all your nutrients from milk and I’d feel, I’d feel bad sort of taking that away from her, but I think when she is weaning I think that’s when I would sort of think about what I am giving her” (P13).

Weaning was considered to be a more suitable window of opportunity to change feeding practices than milk-only diets. However, parents expressed anxieties and a lack of confidence associated with feeding their baby “correctly” and achieving the perceived social expectations of infant feeding.

“Yeah, we are a bit clueless to be honest” (P14).

“…I used to sit and cry over it when she’d gone to bed …” (P10).

Participants reported a lack of information and support around weaning from health professionals and described searching for information and reassurance that they were doing things correctly on the internet or talking to peers.

“Cos the one thing I would say that’s not out there is that you have no idea of what portion size to give your baby they, no one really tells you how much you should be giving a baby” (P6).

### Summary of findings

The study highlights both the barriers and benefits of identifying and communicating the future risk of obesity to parents of infants. Parents believed they would recognise their infant as overweight and despite perceiving they may feel negative as a result, they were receptive to learning about their infant’s future risk. Parents saw risk communication as a cue to action, which is consistent with theoretical models of behaviour change [24]. Parents were concerned about infant overweight and discussed the likely continuation of excess weight from infant to childhood and wanting to prevent this as a reason for wanting risk information. Overfeeding was perceived as the main cause of infant overweight particularly amongst formula-fed babies and parents saw themselves as both responsible and in control of both the development and prevention of excess weight during infancy. Parents’ receptiveness to risk communication and preventative feeding interventions were associated with the developmental milestones of walking and weaning. Walking was seen as a significant milestone for gauging infant overweight, with concerns realised if excess weight persisted. The idea of preventative feeding interventions particularly reduction of infant formula prior to the introduction of solid food was viewed as denying an infant’s needs.

## Discussion

In line with the themes identified, findings are discussed under three main headings: the identification of infant overweight and future risk, the consequences of infant overweight status, and parental attributions of causality, responsibility, and control.

### Identification of infant overweight and future risk

Parents talked openly about overweight and obesity and personalised the issue of overweight when discussing their own infants (subthemes 1a and 1c). Understanding how parents perceive a healthy weight is an important step in the prevention of obesity. In recent years parental perception of childhood weight has been frequently studied and suggests that between 50 and 70% of parents incorrectly perceive their child’s weight status against objective measures of body weight [16, 25, 26]. The existing evidence suggests that factors such as parental overweight and young child age reduce the accuracy parental perception [25]. Parental perception of overweight during infancy poorly understood however, the limited research indicates that parental perception is poor, particularly at around 12 months of age [27]. Parental accounts of how they would recognise infant overweight within this study relied upon subjective observations of infant overweight not objective measures of a measured body weight. Subjective observations of overweight such as comparison to peers and clothing sizes have been previously noted [28]. Mareno argues that current definitions of parental perception of childhood weight are inadequate and lack a conceptual definition and conceptually defines parental perception as, “a parent’s judgement of their child’s body weight” [29]. The study identifies five attributes that formulate perception including, parent recognition of body size, physical appearance, functional abilities, psychosocial effects and health effects of current weight [29], some of which were articulated by parents of this study. The multidimensional nature of parental perceptions discussed by Mareno highlights the pitfalls of relying upon objective measures of weight commonly utilised by health professionals and supports that of Parkinson who suggests the inclusion of subjective measures when talking about weight could improve parental engagement [30].

This study goes further and reveals that despite parents’ perception of an albeit subjective but intuitive recognition of infant overweight they seek reassurance, not censure regarding their parenting practices in relation to feeding and weight (subtheme 3b).

Furthermore, and in support of existing research [12], stage of infant development emerged as meaningful milestone for parents, with the problematisation of infant overweight not given significance until an infant begins walking (subtheme 1c and 3c). Combined with the finding that greater feeding self-efficacy was associated with the introduction of solid food (subtheme 3b), this suggests that there are several windows (rather than a single window) of opportunity for discussion around weight and weight-related behaviours.

### Consequences of infant overweight status

The findings reveal a delay in the point at which parents perceive excessive weight during infancy to be a cause for concern, although continual assurance is sought regarding their infant’s size and weight, (subtheme 1b). Poor perception of the health risks associated with childhood overweight is a possible explanation for lack of parent concern about weight [16]. However, this study revealed that infant weight is characterised as a gateway to future overweight and adult health risks as reported by Eli and colleagues [31]. This might explain the general acceptance of both the validity and utility of risk prediction tools (subtheme 1c). Although health risk and severity perceptions have been associated with prevention in adults [32], this study suggests that for parents of infants the discussion might resonate better if framed in terms of future risk.

This study also emphasises the need to provide parents with a plausible rationale to counter the historical emphasis on under- rather than over-weight [33], which in this study was perpetuated by early interactions with healthcare professionals (subtheme 2a) and common-sense reasoning such low birth-weight infants needing to catch-up on their growth (subthemes 1b).

Although parents perceive their ability to manage or achieve an appropriate or healthy weight in their infants as an indication of good parenting (subtheme 2d), adhering to recommended feeding guidelines were not always seen as compatible with having a contented infant; another, independent marker of good parenting [18] (subtheme 2c). Healthcare professionals need to support parents when they negotiate their goals associated with child’s survival and well-being (in its broadest sense) [34].

### Parental attributions of causality, responsibility, and control

Parents openly claimed responsibility for both causing infant overweight through overfeeding and preventing overweight through healthy feeding practices, implying that it is within their power to control. Nevertheless, parental self-efficacy for behavioural change particularly prior to weaning does not align with an internal locus of control (subthemes 3b and 3c). Parenting self-efficacy is an important determinant of health behaviour change [35] and this should be considered in future studies. Although there is currently no evidence relating specifically to parental self-efficacy and obesity risk in pre-schoolers [36], self-efficacy is strongly associated with parenting competence and child developmental outcomes [37] and recognised as an important factor for successful management of childhood obesity [38].

Inconsistencies exist between lay beliefs of the causes and solutions to obesity described by Ogden and Flanagan (2008) [39] and the framing of obesity as requiring expert intervention [40]. Clearly, parents are poorly served by a situation that they perceive to simultaneously blame and disempower them, and this has important implications for the communication of risk, particularly in relation to non-modifiable factors such as maternal obesity [2]. This likely explains parents’ experiences of, and concerns about, stigma as explained by attribution theory [41, 42]. Indeed, previous research has demonstrated that causal attributions are associated with stigmatisation in relation to preschool children [43].

### Strengths and limitations

A key strength of this study is the recruitment of parents of an unhealthy body weight with over 50 % reporting measures resulting in a Body Mass Index (BMI) indicative of overweight or obese. One major limitation of the research findings is the hypothetical nature of some of the research questions. In many cases participants voluntarily personalised the questions to be in relation to their own infant, however when discussing the anticipated consequences and benefits of being told about future obesity risk responses were hypothetical. It is also reasonable to assume that parents self-selected for the study on the basis that the topic of childhood overweight was salient to them and this may have influenced the findings.

## Conclusion

Parents in this study reported high levels of personal responsibility around the development of overweight, through overfeeding and believed they could prevent of overweight by changing infant feeding practices. These findings demonstrate the importance of non-judgmental communication skills and appropriate support to ensure risk communication does not result in feelings of shame, self-blame and the disempowerment of parents. Parents demonstrated a hypothetical interest in learning about their infant’s future risk of overweight particularly once their infant is able to walk. Although the parental response to risk communication was positive, a preference for intervention at 1 year would mean that opportunities to change infant feeding practices, in particular responsive feeding would be missed. This current mismatch between the optimum evidence-based point for early obesity prevention efforts and the point perceived as acceptable by parents suggests the need to strike a balance. Further exploration and confirmation with the use of an experimental study design is required to establish the outcomes of risk communication with parents of infants at different ages. Alongside this, practical strategies are needed to enhance parental understanding of rapid weight gain via improved clinical explanation and the use of subjective measures. Also paramount is reassurance that challenges historic perceptions and parental fears surrounding underweight during infancy.

## Abbreviations

BMI: Body Mass Index
IMD: Index of Multiple Deprivation
NCMP: National Child Measurement Programme
